# An Experimental *Toxoplasma gondii* Dose Response Challenge Model to Study Therapeutic or Vaccine Efficacy in Cats

**DOI:** 10.1371/journal.pone.0104740

**Published:** 2014-09-03

**Authors:** Jan B. W. J. Cornelissen, Joke W. B. van der Giessen, Katsuhisa Takumi, Peter F. M. Teunis, Henk J. Wisselink

**Affiliations:** 1 Central Veterinary Institute of Wageningen UR, Department of Infection Biology, Lelystad, The Netherlands; 2 Central Veterinary Institute of Wageningen UR, Department of Bacteriology and TSEs, Lelystad, The Netherlands; 3 National Institute of Public Health and the Environment (RIVM), Centre for Zoonoses and Environmental Microbiology, Bilthoven, The Netherlands; 4 National Institute for Public Health and the Enviroment (RIVM), Centre for Epidemiology, Bilthoven, The Netherlands; University of Wisconsin Medical School, United States of America

## Abstract

High numbers of *Toxoplasma gondii* oocysts in the environment are a risk factor to humans. The environmental contamination might be reduced by vaccinating the definitive host, cats. An experimental challenge model is necessary to quantitatively assess the efficacy of a vaccine or drug treatment. Previous studies have indicated that bradyzoites are highly infectious for cats. To infect cats, tissue cysts were isolated from the brains of mice infected with oocysts of *T. gondii* M4 strain, and bradyzoites were released by pepsin digestion. Free bradyzoites were counted and graded doses (1000, 100, 50, 10), and 250 intact tissue cysts were inoculated orally into three cats each. Oocysts shed by these five groups of cats were collected from faeces by flotation techniques, counted microscopically and estimated by real time PCR. Additionally, the number of *T. gondii* in heart, tongue and brains were estimated, and serology for anti *T. gondii* antibodies was performed. A Beta-Poisson dose-response model was used to estimate the infectivity of single bradyzoites and linear regression was used to determine the relation between inoculated dose and numbers of oocyst shed. We found that real time PCR was more sensitive than microscopic detection of oocysts, and oocysts were detected by PCR in faeces of cats fed 10 bradyzoites but by microscopic examination. Real time PCR may only detect fragments of *T. gondii* DNA without the presence of oocysts in low doses. Prevalence of tissue cysts of *T. gondii* in tongue, heart and brains, and anti *T. gondii* antibody concentrations were all found to depend on the inoculated bradyzoite dose. The combination of the experimental challenge model and the dose response analysis provides a suitable reference for quantifying the potential reduction in human health risk due to a treatment of domestic cats by vaccination or by therapeutic drug application.

## Introduction

Toxoplasmosis, caused by the protozoan parasite *Toxoplasma gondii* has a worldwide distribution, one-third of the global human population has been exposed to this parasite [1]. The integrated public health impact defined as disease burden expressed in Disability Adjusted Life Years (DALYs) is globally considered to be very high [2]. In the USA, *T. gondii* ranked third out of 14 foodborne pathogens [3] and in the Netherlands, the total burden of toxoplasmosis was estimated 3620 DALYs, ranking *T. gondii* as the first among 14 enteral pathogens examined [4]. Because of this high public health impact of toxoplasmosis intervention measures need to be implemented.

Cats and other Felidae are the primary source of a *T. gondii* infection [5], [6]. Cats become infected by feeding on infected meat (wild rodents and birds) or, less effectively, by ingestion of sporulated oocysts [7]. This results in an, for cats only enteroepithelial sexual cycle that leads to the shedding of millions of oocysts into the environment within a period of two to three weeks [8], [9], [10], [11]. The oocysts may contaminate the environment and can resist extreme environmental conditions, remaining infectious for periods up to 18 months [12] or longer [13], [14].

Toxoplasma may be transmitted to humans through the consumption of raw or undercooked meat from livestock (e.g. pigs, cows and sheep) containing tissue cysts [15] or by ingesting of food or water contaminated with oocysts from infected cat faeces [16], [17]. *T gondii* can also be transmitted vertically by a primary infection with *T. gondii* during pregnancy and entering of the parasite into the foetal circulation by infection of the placenta [18].

Since *T. gondii* can be considered a major foodborne pathogen, the development of strategies to prevent humans to become infected is of increasing importance. The ultimate control strategy would be to prevent infected cats from shedding oocysts in the environment. Although vaccines nor other drug treatments in cats are yet commercially available, such control strategies may become available in the future [19], [20], [21].

To evaluate the efficacy of vaccines or drugs, a standardised animal model is needed. Cats can be experimentally infected with tissue cysts [22] whereby only a few tissue cysts are necessary to infect cats [23]. Isolated bradyzoites from tissue cysts are also very infectious for cats [25], [26], [27], after ingesting a few bradyzoites cats can shed millions of oocysts [25].

Several studies have been published studying *T. gondii* vaccine development in cats [28], [29], [30], [31], [32], [33], [34]. In these studies, vaccines were evaluated using 200 to1000 brain tissue cysts produced in mice to challenge orally cats. However, a tissue cyst may contain 2 to 1,000 bradyzoites [24] indicating that the dose is not well defined when tissue cysts are used to infect cats.

It was our aim to develop a standardised challenge model in cats. Cats were experimentally infected with various doses of bradyzoites harvested from tissue cysts of experimentally infected mice. The results were used to estimate the infectivity, by means of a dose response model, appropriate for challenge studies in cats. We found that shedding of oocysts by cats after experimental infection is dose- and time-dependent.

## Materials and Methods

### 2.1. Toxoplasma strain

Oocysts from *T. gondii* genotype II strain M4 were obtained from Prof. Dr. E.A. Innes of the Moredun Research Institute (Edinburgh, Scotland).

### 2.2 *T. gondii* infection in mice: preparing of inoculum for infection of cats

To prepare tissue cysts and bradyzoites for experimentally infecting cats, Swiss Webster mice at the age of six weeks were orally infected with 100 oocysts of *T. gondii* M4 strain in a volume of 0.25 ml PBS. At seven weeks p.i. three mice were sacrificed, brains were collected and brain tissue cysts were harvested by a discontinuous 30–90% Percoll gradient [35] according to the modified protocol of Fritz et al. [27]. Briefly, three-quarters of each brain were passed through a 100 µm cell strainer into a 50 ml conical tube. The plunger of a six ml syringe was used to press the brain tissue through the strainer, thereby retaining the fatty tissue in the strainer. Brain tissue was washed with PBS and resuspended in a volume of 4 ml. A density gradient was prepared for each sample in a 50 ml conical tube by carefully layering (from bottom to top) 9 ml 90% Percoll, followed by 9 ml 30% Percoll and finally followed by 10 ml brain suspension. Percoll dilutions were made using one×PBS. Gradient preparations were centrifuged at 1200× g for 15 min at 4°C. Tissue cysts were harvested from the 30% and 30%/90% interface, suspended in 45 ml PBS and centrifuged at 3000× g for 15 min at 4°C. The supernatant was removed and the pellet containing the tissue cysts was transferred to a 1.5 ml micro centrifuge tube. The volume was brought up to one ml with Hank's Balanced Salt Solution (HBSS; Life Technologies Europe BV Bleiswijk, the Netherlands). A 25 µl aliquot was used for tissue cyst enumeration under light microscopy at a 40× magnification. To release the bradyzoites from the tissue cysts an equal volume of pepsin digestive fluid (0.26 g pepsin; 5 g NaCl; 7 g HCl; distilled water to 500 ml) was added to the tissue cysts (final concentration 0.026%) [36]. Subsequently, the suspension was incubated at 37°C for 10 min, neutralized with Na_2_CO_3_ and suspended in Dulbecco's modified Eagle's medium (DMEM) plus 3% foetal calf serum (FCS). Bradyzoites were counted in a haemocytometer and adjusted to a concentration of 2.5×10^3^/ml.

To prepare bradyzoites and tissue cysts for infection of cats, ten mice were orally inoculated with 100 oocysts of *T. gondii* strain M4. At seven weeks p.i. three mice were sacrificed. Per mouse brain 80–1200 tissue cysts were collected and per tissue cyst 166–275 bradyzoites were collected. To infect cats with bradyzoites, a stock solution of 2.5 * 10^3^ bradyzoites per ml was prepared and further diluted preparing inoculation doses of 1000, 100, 50, and 10 bradyzoites in a volume of 2.5 ml. To infect cats with tissue cysts a solution of 250 tissue cysts in a volume of 2.5 ml was prepared.

### 2.3 *T. gondii* infection in cats

Fifteen cats (10 male and 5 female) at the age of 11 weeks were obtained from Isoquimen, Sant Feliu de Codines, Spain. In a health monitoring report, the supplier of the cats declared that the cat colony is free for *T. gondii* as determined by blood serum testing for anti *T. gondii* antibodies and examination of faeces for the presence of oocysts. For verification of absence of *T. gondii* infection, blood serum of the mothers (n = 8) of the kittens was obtained in advance of delivery of the kittens and tested for anti *T. gondii* antibodies by an indirect ELISA with tachyzoite antigen as described below. After transport to the animal facilities of CVI (Lelystad, The Netherlands), the kittens received commercially available dry food and tap water ad libitum. Kittens were housed individually in an accommodation according to EU regulation 2007/526/EC. General health observations were conducted daily during cleaning and feeding activities and during social interaction with biotechnicians. Kittens were acclimatized to the animal experimental facility for seven days. During the acclimatization period, oocysts counts were performed in faeces as described below to demonstrate the absence of a *T. gondii* infection. At the age of 12 weeks, five groups of three kittens were orally challenged with 1000, 100, 50 and 10 bradyzoites of the *T. gondii* M4 strain in 2.5 ml DMEM-3% FCS using a curved blunt cannula. One group of three SPF kittens was orally challenged with 250 brain tissue cysts from mice infected with *T. gondii* M4 strain in 2.5 ml PBS. Between 3 and 21 days after inoculation, all faeces were collected daily from a litter box filled with grit. From each kitten, all faeces was sieved out, collected and sent to the laboratory. The litter box was cleaned thereafter.

From each kitten, faeces was weighed and oocysts were purified from 2 gram out of 25 gram mixed faeces by 100 µM sieving, and saturated NaCl (d = 1.18 g/ml) flotation according to Wainwright et al. [37]. Oocysts were floated by centrifugation at 2500 g for 10 minutes (non-braked). A sample of 200 µl with floated oocysts was taken by carefully pipetting from the uppermost part of the contents of the centrifuge tube. In this sample the number of floated oocysts per gram (OPG) was counted using a haemocytometer and the quantity of *T. gondii* DNA was determined by real time PCR for 529 *T. gondii* gen as described below. Determination of oocyst shedding was finished when no oocysts had been detected for three consecutive days. Serum samples were collected from a jugular vein of the kittens at the day of challenge and at one, two, and three weeks post-challenge (p.c.). At three weeks post challenge, when no more shedding occurred the experiment was terminated. Kittens were brought under full narcosis with 40 mg/kg Ketamine (Alfasan, Woerden, The Netherlands) plus 2 mg/kg Xylazine (Eurovet, Putten, The Netherlands) and 0.05 mg/kg Atropine (Eurovet, Putten, The Netherlands). Subsequently, kittens were bled and from each kitten, samples were obtained from brains, tongue and heart for detection of *T. gondii*. A quarter of the brains of each kitten was passed through a 100 µm cell strainer into a 50 ml conical tube using the plunger of a six ml syringe to press the tissue through the strainer (retaining the fatty tissue in the strainer) and washed with PBS to a total volume of four ml. Three-quarters of the tongue and heart of each kitten were homogenized in four ml PBS with a disposable Omni Tissue homogenizer during one minute. A 25 µl aliquot was taken for cyst determination under light microscopy at a magnification of 40× and an aliquot of 200 µl was taken for DNA extraction.

### 2.4 DNA extraction and real time PCR conditions

**Figure 1:**
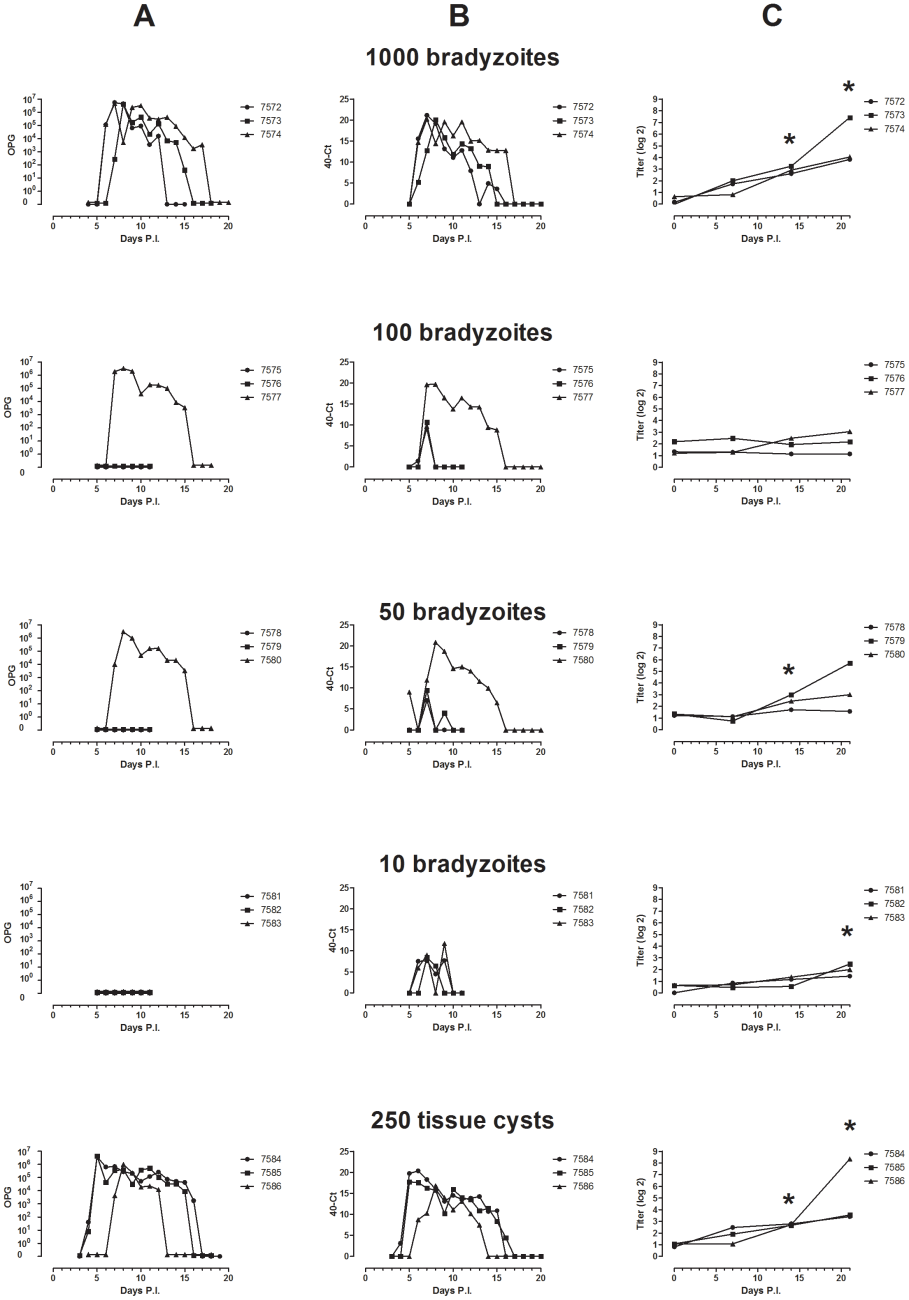
Oocysts shedding (A), real time PCR values of the oocysts shedding (B) and antibody response (log2 titres) (C) from the kittens infected with 10, 50, 100 and 1000 bradyzoites or 250 tissue cysts of T. *gondii* Strain M4. The groups contained three cats each. *P<0.05 significant to the anti *T. gondii* IgG response at week 0.

For detection of *T. gondii* by real time PCR, 200 µl floated oocysts preparations from faeces of cats, heart, brain and/or tongue tissue samples of kittens were further homogenized by vortexing the samples with 50–100 glass beads (0.4 mm) for one to two minutes. Microscopically it was confirmed that after this treatment most oocysts and tissue cysts had been broken. DNA was extracted with the Qiagen DNA easy Blood & Tissue Kit (Qiagen GMBH, Hilden, Germany) according to the manufacturer's manual. One µl DNA was tested by the PCR with on-line detection using Syber Green PCR Master Mix (Applied Biosystems, Foster City, CA, USA) in an ABI 7500 Real-Time PCR system (PE Applied Biosystems, Foster City, CA, USA). A standard reaction mixture contained 12.5 µl of two× QuantiTect SYBR Green PCR Master Mix, one µl (10 µM) of the primers, one µl of DNA template and 10.5 µl PCR grade water. The Tox-oligonucleotides are complementary to the 529-bp repeat element (GenBank AF146527) [38] and the forward and reverse Toxoplasma primers were described previously as Tox-9, and Tox-11 [39]. The cycling profile involved an initial PCR activation step at 95°C for 10 min, followed by 40 cycles of denaturation at 94°C for 15 s, primer annealing at 59°C for 30 s and extension at 72°C for 30 s. The fluorescence was measured at the end of each cycle. Following amplification, a melt curve analysis was performed to verify the specificity of the amplified products by their specific melting temperatures (Tm). Post amplification melting curve with SYBR Green dye of Toxoplasma positive DNA samples revealed one peak. For quantification of the amount of Toxoplasma DNA in the samples, a standard curve of a plasmid containing the 529 repeat gen in pGEM-T easy (Promega Benelux b.v. Leiden, The Netherlands) was used. Amounts of DNA were based on the log 10 dilutions of the standard. All samples analysed had a blank PCR control and samples negative for Toxoplasma DNA are depicted in Fig. 1B as 40-Ct = 0, whereas samples with 40-Ct>0 were considered as positive. Data acquisition and analysis of the results were performed using the 7500 System SDS Software Version 2.0.1 (Applied Biosystems).

### 2.5 Serology for *T. gondii*

Serum samples of the infected cats were processed with an in-house developed ELISA test for determining anti *T. gondii* antibody titers. ELISA plates (Nunc Polysorp 475094; Sanbio, Uden, The Netherlands) were coated with a *T. gondii* tachyzoites lysate (5 µg/ml in Na_2_CO_3_ buffer pH 9.5) overnight at 37°C. Wells were incubated for one h at 37°C with sera in two-fold dilutions from 1/25 to 1/3200 in PBS containing 0.05% Tween-80. The next incubation was for one h at 37°C with a peroxidase-conjugated Goat anti Cat-h&l (clone 10MG1-2, Bio connect, Huisen, The Netherlands) HRPO conjugate in a dilution of 1∶10.000 in ELISA buffer. Serum from cats either infected or free from a *T. gondii* infection were used as positive and negative control. As substrate, 3,3′,5,5′-tetramethylbenzidine (TMB) with H_2_O_2_ was used. After incubation for 5 min at room temperature, the reaction was stopped by the addition of sulphuric acid. The plate was read by the use of a Bio-Kinetics ELISA reader (Bio-Tek Instruments Inc., Winooski, VT) at 450 nm. Antibody titres were expressed as the 2-log of the regression coefficient of the optical density vs. serum concentration. Per group of three cats, ELISA results were taken to calculate the geometric means and standard deviations. Antibody titres (2-log) were compared to antibody titre in serum samples at day 0, and were analysed for statistical significance by the Mann-Whitney U test in the GraphPad Prism version 5.0 software, and where considered significant if the P-value<0.05.

### 2.6 Dose-Response Model

The infection status of cats inoculated with *T. gondii* M4, as judged by OPG count >0 was fitted by a Beta-Poisson dose-response model [40]. This model is based on the assumption that any single viable bradyzoite may cause infection, and accounts for variability in the probability of infection resulting from variation in host susceptibility or pathogen infectivity, due to genetic or other factors. Sampling effects in the applied inoculum were accounted for by assuming that the ingested number of bradyzoites is a Poisson sample from a suspension with mean equal to the target dose. Given a certain probability of infection at each dose, the numbers of infected cats at that dose are binomially distributed. Using the binomial likelihood the two parameters (α, β) can be estimated. A Monte Carlo sample of dose response parameters was obtained based on the oocysts output using JAGS (3.2.0), a program for analysis of Bayesian hierarchical models using Markov chain Monte Carlo (MCMC) analysis (http://mcmc-jags.sourceforge.net).

### 2.7 Animal Ethics Committee

The animal study has been conducted with permission of the Animal Ethics Committee of the Animal Sciences Group (Lelystad, the Netherlands) dated 3 October 2012, registered under number 2012073.c.

## 3. Results

### 3.1 *T. gondii* infection in kittens

None of the cats showed any clinical symptom (depression, central nervous behaviour, gastro intestinal signs, diarrhoea) throughout the study period.

#### Oocysts in faeces

The dynamics of oocyst shedding by cats after challenge infection with mouse bradyzoites or tissue cysts of *T. gondii* strain M4 is shown in Table 1. All three kittens from the group infected with 250 tissue cysts shed oocysts in the faeces from day 4 till day 16 (Fig. 1A). The three kittens infected with 1000 bradyzoites shed oocysts from day 6 till day 17. At peak shedding on day 6–7 p.i, the kittens in this group produced 5* 10∧6 oocysts per gram faeces (OPG). From the kittens infected with 100 or 50 bradyzoites only one out of three shed oocysts at day 7–16 p.i., and from the kittens infected with 10 bradyzoites none shed oocysts, as determined microscopically.

**Table 1:** The dynamics of oocyst shedding by cats after challenge infection with mouse bradyzoites or tissue cysts of *T. gondii* strain M4.

Experimental group	Dose	Stage	No of cats	No. of cats positive		Days after infection						Total of oocysts shed	
						Mean pre-patent period		Mean patent period		Mean peak oocyst shedding			
				ME**	Real Time PCR	ME	Real Time PCR	ME	Real Time PCR	ME	Real Time PCR	ME	Real-Time PCR**
1	10	Bradyzoite	3	0	3	NA*	5.3	NA*	2.7	NA*	6.7	0	1.3E-07
2	50	Bradyzoite	3	1	3	7	6	9	4.3	9	7	1.9E+07	4.3E-05
3	100	Bradyzoite	3	1	3	7	5.7	9	4	8	7	6.9E+07	9.0E-05
4	1000	Bradyzoite	3	3	3	5.3	5	9.3	10	7.3	7.3	2.5E+08	4.0E-04
5	250	Tissue cysts	3	3	3	5	4	10.3	10.7	6	7	9.1E+07	1.0E-04

*Not Applicable.

**Microscopical examination.

#### Real time PCR of faeces

All kittens which tested positive by microscopic examination were also positive by real time PCR. Moreover, all OPG-negative animals: the two negative kittens infected with 50 or 100 bradyzoites, and all kittens challenged with 10 bradyzoites became also positive for *T. gondii* by real rime PCR (Fig. 1B). The Ct values in these lower dose groups were higher showing that the concentration of available DNA was lower.

#### Serology

Compared to kittens in the other groups, the kittens that were infected with 250 tissue cysts or 1000 bradyzoites had the highest titres (Fig. 1C). In both groups, antibody titres at 14 and 21 days p.c. appeared to be significantly higher (*P*<0.05) compared to day 0. In the other dose groups the increase was much smaller and in the kittens infected with 10 or 50 bradyzoites we found a significant increase in IgG titre (*P*<0.05) respectively at week three and two compared to antibody titre in serum samples obtained at day 0. One kitten (nr 7579) of the group infected with 50 bradyzoites showed an IgG titre of 5.7 (2log) at day 21 p.c., shedding of oocysts in faeces of this cat was only confirmed by real time PCR and not microscopically.

#### Real Time PCR and microscopy of brain, tongue and heart

The real time PCR results detected *T. gondii* DNA in five tongue and in seven heart samples of the infected kittens. Of all the brain samples tested, only one kitten (100 bradyzoites) had very low Ct values in the real time PCR on brain samples, the other 14 kittens were negative (Table 2). Ct values after PCR testing of heart and tongue of the cats infected with 1000 bradyzoites were comparable, indicating that there was no difference in distribution of tissue cysts over the tongue, and heart. (Table 2). Microscopically, we did not find any tissue cysts in brain, heart and tongue samples.

**Table 2:** Prevalence of tissue cysts as revealed by real time PCR of the heart, tongue and brains of cats infected with bradyzoites or tissue cysts of *T. gondii* strain M4.

Kitten no.	Dose	Infectious Material	40-Ct value		
			Heart	Tongue	Brains
7581	10	Bradyzoites	0	0	0
7582	10	Bradyzoites	0	0	0
7583	10	Bradyzoites	0	0	0
7578	50	Bradyzoites	0	0	0
7579	50	Bradyzoites	9.0	13.4	0
7580	50	Bradyzoites	0.2	0.0	0
7575	100	Bradyzoites	0	0.0	0
7576	100	Bradyzoites	0	0.0	4.1
7577	100	Bradyzoites	5	11.7	0
7572	1000	Bradyzoites	0	12.8	0
7573	1000	Bradyzoites	11.6	14.4	0
7574	1000	Bradyzoites	12.2	15.5	0
7584	250	Tissue cysts	3.2	0	0
7585	250	Tissue cysts	0	0	0
7586	250	Tissue cysts	11.7	0	0

### 3.2 *T. gondii* dose response model in cats

**Figure 2:**
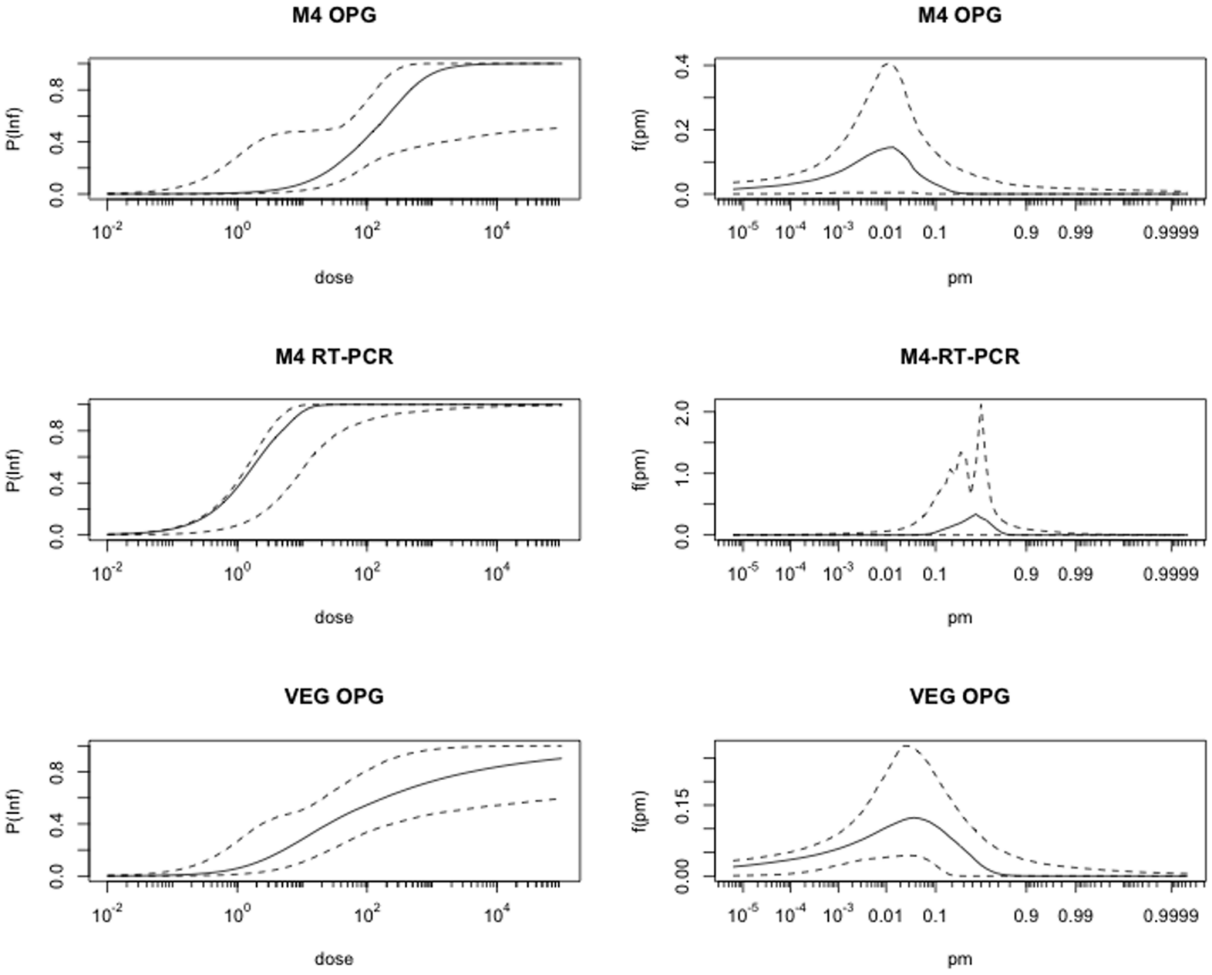
Panels in column A illustrate the dose-response for *Toxoplasma gondii* in cats: Horizontal axes show mean number of bradyzoites; vertical axes show probability that a cat is infected following ingestion. Each panel refers to: OPG of M4 strain (top), real time-values of M4 strain (middle) and OPG of VEG strain (bottom). Panels in column B illustrate posterior density for the dose response parameter (probability of infection by a single bradyzoites): Horizontal axes show possible estimate for the parameter; vertical axes show probability density. Solid line indicates the mean and dashed lines are 2.5% and 97.5% percentiles.

**Figure 3:**
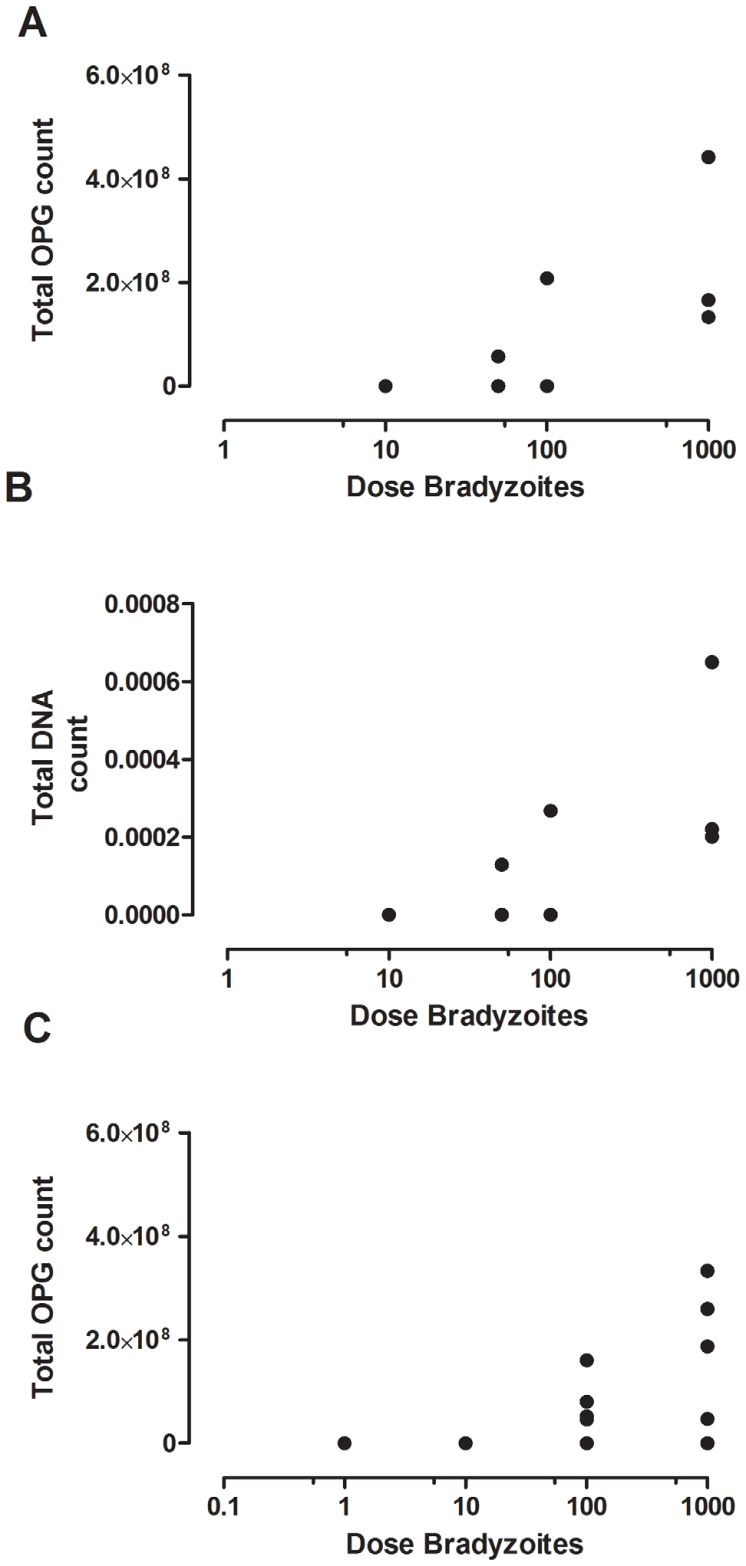
Total amount of *T. gondii* shedding per individual infected cat: M4 strain based on the OPG faecal output (A), the real time PCR DNA values in the faeces (B), the VEG strain (C) based on the OPG faecal output.

Infection was defined as: 1. counting one or more oocysts in faecal samples (oocysts per gram, OPG), and 2. positive results of the real time PCR of faecal samples. The estimated mean ID50 based on the OPG counts is 181 bradyzoites (36–806 bradyzoites, 95% credible interval (ci), Fig. 2A). The estimated probability of infection per single bradyzoite is 0.02 (95% ci 0.003–0.13 per single bradyzoites, Fig. 2B). Based on the infection as determined by real time PCR, the estimated mean ID50 is two bradyzoites with a 95% credible interval of 0.044–11 bradyzoites. Equivalently, the probability of infection per single bradyzoite is 0.38 (95% ci 0.08–0.52 per single bradyzoite). To investigate whether infectivity is specific to the M4-strain, we also applied this dose response model to a similar experimental challenge model in which the VEG strain was used (Fig. 2C; the datasets of experiment I and II from Dubey [25]). Using OPG>0 to characterize infection, the estimated mean ID50 was 214 bradyzoites (95% ci 7–1800 bradyzoites). To investigate whether the estimated infectivities of the M4 and VEG strains (Fig. 2A; M4 and VEG OPG) are different, a likelihood ratio test was done, showing that a model assuming equal infectivities of M4 and VEG did not provide a significantly worse fit than the two separate infectivity estimates (*P*-value = 0.47: likelihood ratio test, 2 d.f.). The total OPG and DNA count shed by cats in the experimental groups using 10, 100 and 1000 bradyzoiets are shown in Fig. 3A and B. In addition, the total OPG counts shed by cats in the experimental infections as described by Dubey [25] are shown in Fig. 3C indicating that total OPG shedding was indeed in the same order of magnitude for the two studies.

## Discussion

The aim of our study was to establish a standardised challenge model in cats as a reference for studying the effectiveness of vaccines or therapeutic drugs against T. *gondii* infection in cats.

We selected *T. gondii* strain M4. This strain belongs to genotype II, the most predominant *T. gondii* genotype in Europe [41]. Strain M4 is able to produce brain tissue cysts in mouse and oocysts in the gut of cats [27]. Strain M4 is therefore a suitable candidate for a standardised challenge model in cats.

To prepare the challenge inoculum to experimentally infect cats, a sufficiently concentrated suspension of fresh bradyzoites and tissue cysts was produced in mice, seven weeks after oral infection with oocysts. This finding is in agreement with other studies [22] in which harvesting tissue cysts after 6–8 weeks is sufficient time for infectious stages to develop.

We inoculated cats at an age of 12 weeks orally with four different doses (from low to high) of bradyzoites and determined a dose-response model for *T. gondii*. None of the challenged cats had clinical symptoms. This is in agreement with previous findings that cats older than three months rarely develop clinical signs after oral infection with tissue cysts [22].

We found that cats can shed millions of oocysts in their faeces after ingesting as few as 50 bradyzoites. These results are in agreement with results obtained by Dubey [25] who found that 100 bradyzoites of *T. gondii* strain VEG orally inoculated could lead to shedding of oocysts in the faeces. Our findings confirm the high infectivity of the bradyzoite stage of the parasite for cats [25], [26], [27]; therefore this parasite stage is an important candidate for use as challenge inoculum in vaccination or treatment-challenge studies in cats. Other authors [26], [29], [32], [34] used mouse tissue cysts to infect cats, however as found by others and confirmed in this study, tissue cysts may contain five to several hundreds of bradyzoites, “depending on the age of the cysts” [24]. Suspended bradyzoites in contrast to tissue cysts provide a reproducible dose. Using the different *T. gondii* strain M4 bradyzoites doses in cats, we found a dose response relation. In addition, there was no significant difference in the dose response between the *T. gondii* M4 strain used in this study and the *T. gondii* VEG strain as reported by Dubey [25] using OPG as a response. Concerning M4 strain only, a dose response relation shifted toward higher infectivity when real time PCR positivity instead of OPG is chosen as an endpoint. Real time PCR and microscopy both detect oocyst shedding, but the 529 repeat element detected by real time PCR is present in 200–300 copies per parasite and is therefore more sensitive [38]. At low doses (100 – 50 bradyzoites), real time PCR detects more positives, and even in the lowest dose group (10 bradyzoites) the real time PCR detects low levels of shedding of *T. gondii* genomes. Interestingly, these low dose shedding responses appear different from those in OPG positive animals. The Ct values are 10–15 cycles lower than those in OPG positive, and the duration of shedding is 5–10 days shorter. The question is whether this low-level shedding represents true infection, producing infectious oocysts. The real time PCR assay may detect the DNA extracted from infectious oocysts in all dose groups (as confirmed with OPG shedding) or it may only detect fragments of *T. gondii* DNA without the presence of oocysts in low dose groups. This might have consequences in case of challenge studies to evaluate the vaccine effectiveness for example, since only infectious oocysts shed by cats are a risk for humans and are thus of epidemiological relevance.

In previous studies, it was described that pre-patent periods in cats differs from 3 to10 days after the ingestion of tissue cysts (bradyzoites) and 18 days or more after the ingestion of oocysts or tachyzoites [23], [42], [43], [44]. Our study confirmed that the prepatent period was 5–7 days among all bradyzoite challenge doses. The patent period increased with the increasing dose of bradyzoites. Hence, it is an important feature to determine the optimal dose for vaccination challenge or therapy experiments.

It has been described that IgG antibodies are initially absent during primary *T. gondii* infection in cats, but then start to increase after a few weeks to reach protective levels and then remain detectable for years. IgM antibodies rise within days, and usually decrease over the following few weeks. Dubey et al. [45] reports that cats seroconverted 10 days p.i and high titres persisted even after 6 years, however, in some cats with a chronic infection IgM persists. In the present study we detected significantly higher IgG titres as early as three weeks after infection in cats that received the highest dose of *T. gondii* bradyzoites and the tissue cysts challenge group.

We detected *T. gondii* DNA by real time PCR in tongue, heart and in one brain tissue of infected cats. Dubey described earlier the detection of *T. gondii* tissue cysts in heart, skeletal muscles, diaphragm, brain, spleen, kidneys; pancreas, stomach, adrenals, lungs, thymus, cervical and mesenteric lymph nodes salivary glands and eyes and tongue of bradyzoites infected cats [26], of which tongue, heart and brain were important target organs [46].The cats with the highest infection dose revealed a high *T. gondii* DNA load in tongue and heart.

In summary, we conclude that the oocysts of *T. gondii* strain M4 can be used to produce high numbers of bradyzoites in the brain of the mouse to be used as a challenge model in cats. Moreover, the bradyzoite stage of *T. gondii* strains can be used to establish a dose response model for oral challenge with *T. gondii* in the cat. The dose response in cats is *T. gondii* strain independent at least for the M4 and VEG strains. However, the results using microscopy or real time PCR will have influence on the outcomes of the dose response, most likely caused by differences in detection limit of each assay. Both the probability of infection and the numbers of oocysts shedded by infected cats appeared to depend on the challenge dose. Therefore, we can use this M4 challenge model quantitatively to determine the effects of any intervention, vaccine or drug induced, expressed in a reduction of the total oocyst output of *T. gondii* clonal types II and III infected cats. For the future, our *T. gondii* dose response model in cats can be exploited to evaluate different strategies aiming at cats to reduce oocysts shedding.
